# Highly efficient photodegradation of raw landfill leachate using cost-effective and optimized g-C_3_N_4_/SnO_2_/WO_3_ quantum dots under Vis–NIR light

**DOI:** 10.1038/s41598-022-24143-3

**Published:** 2022-11-14

**Authors:** Amirmohsen Samadi, Mohammad Delnavaz, Ali Rashtizadeh, Nima Heidarzadeh

**Affiliations:** Faculty of Engineering, Civil Engineering Department, Kharazmi University, Tehran, 15719-14911 Iran

**Keywords:** Environmental sciences, Environmental chemistry

## Abstract

In this study, photodegradation of raw landfill leachate under Vis–NIR irradiation and sunlight has been investigated using optimized g-C_3_N_4_/SnO_2_/WO_3_ quantum dots as a novel nanocomposite. g-C_3_N_4_/SnO_2_/WO_3_ QDs was successfully synthesized and characterized using various analyses. The best mixing ratios of the nanocomposite components were obtained by response surface methodology (RSM). The morphology and the surface area characteristics of the photocatalyst were investigated by scanning and transmission electron microscopy (SEM and TEM) and Brunauer, Emmett and Teller (BET) analysis. Results of UV–Visible diffuse reflectance spectroscopy (UV–Vis DRS) and photoluminescence (PL) spectrum revealed that the nanocomposite has a great light absorption capacity and improved separation of charge carriers. Using the optimized nanocomposite with the best mixing ratios of urea, SnO_2_, and WO_3_ QDs solution, obtained from the central composite design (CCD), the chemical oxygen demand (COD) of the leachate (4575 mg/L) was reduced by 74% and 47% in 4 h under visible-NIR and sunlight irradiations, respectively. Gas chromatography–mass spectrometry (GC–MS) analysis also revealed that a significant reduction of aromatic compounds of the raw leachate occurred after the photodegradation process with g-C_3_N_4_/SnO_2_/WO_3_QDs nanocomposite. Moreover, the stability and recyclability of the photocatalyst were evaluated, and it was observed that after five experimental cycles of leachate degradation, no significant loss of nanocomposite performance could be seen. Financial analysis was also performed, and the feasibility of this process was investigated.

## Introduction

Treatment of landfill leachate is a crucial criterion for sustainable development and environmental conservation^1^. Landfill leachate contains many organic pollutants such as alcohols and fatty acids, inorganic contaminants like NH_4_-N and heavy metals, and pathogens. Therefore it is categorized as one of the recalcitrant wastewaters^2,3^. Effective treatment of landfill leachate is a key part of waste management, which remains a challenge for many developing cities and countries and is necessary for building livable and sustainable cities^4^. In addition to serious health problems, entering such liquid, which contains various pollutants, into groundwater and surface water, can cause significant economic management and financial issues^5,6^. The importance of this issue is even greater in a country like Iran which is facing a water crisis^7^.

In recent years, g-C_3_N_4_ (denoted as CN) has been widely investigated by researchers as a promising photocatalyst^8^. CN is a type of low-cost and non-toxic organic semiconductor with a small 2.7 eV band gap, non-metal nature, strong visible light absorption, convenient band structures, and high physical and chemical stability, which make it a great choice to use in the photocatalytic treatment of wastewaters. Moreover, the synthesis of CN photocatalyst is facile and inexpensive^9^. However, bare graphitic carbon nitride suffers from limitations like fast recombination of photogenerated charge carriers, which causes low efficiency of photodegradation of pollutants, and small specific surface area, which leads to a reduction in the adsorption of harmful organic contaminants and absorption of visible radiation^10^. To overcome this drawback, a variety of techniques have been extended, such as coupling with some semiconductors like TiO_2_, ZnO, SnO_2_, and WO_3_^11–13^.

SnO_2_ as an *n*-type semiconductor (energy band gap: 3.6 eV) has received significant attention due to its low cost, abundance, high photosensitivity, facile fabrication, good transparency, stability, and environmental friendliness^14–17^. Despite these advantages, the applied usage of SnO_2_ as an effective photocatalytic semiconductor is limited because of its rapid photoinduced electron–hole recombination and low photocatalytic efficiency under visible light^18,19^. However, SnO_2_ has been widely used to combine with different semiconductors like Fe_2_O_3_, CuO, TiO_2_, ZnO, SnO, and ZnS because of its relatively proper conduction band potential of 0.5–1.0 eV, which makes it an excellent choice to be used as an electron acceptor to make hybrid composites and promote separation of electrons and holes under visible light^20–23^. Combining SnO_2_ and CN with a relatively negative conduction band potential of − 1.0 to − 1.5 eV leads to improvement of photocatalytic efficiency due to forming systems with correlated band structures. This combination could solve the problems of both semiconductors and possesses a great specific surface area with a reduced recombination rate of electrons and holes^24^.

Among all metal oxides, tungsten trioxide, as an inexpensive n-type semiconductor with a small bandgap of 2.6 eV, has been considered a favorable photocatalyst due to its unique optical, thermal, electrical, and physicochemical properties^25^. It is because of these properties that WO_3_ is utilized in several applications, including fuel cells, sensors, hydrogen generation, adsorption of contaminants, and especially, photodegradation of organic pollutants in the visible light region^26,27^. Combining WO_3_ and g-C_3_N_4_ can lead to forming an effective photocatalyst with properties that can help to overcome the problems of traditional photocatalysts. Therefore, much research has been done to assess the effectiveness of WO_3_/g-C_3_N_4_ in the photodegradation of wastewater pollutants. For instance, Chen et al., Huang et al., and Meng et al. synthesized WO_3_/g-C_3_N_4_ composite and utilized them in photodegradation of methyl orange, methylene blue, and RhB, respectively^28–30^. They realized that its photocatalytic performance is promising and better than single WO_3_ and g-C_3_N_4_. On the other hand, WO_3_ quantum dots exhibit superior unique properties compared to normal WO_3_ nanoparticles. WO_3_QDs can desirably separate photogenerated electron–hole pairs, trap photogenerated electrons easily, and it is a favorable receiver for conducting electrons^31^. Moreover, the catalytically active sites in 1D WO_3_ nanowires and 2D WO_3_ nanosheets have fewer defects than those in WO_3_ quantum dots. It is because of the quantum size effects and abundant surface defects of WO_3_QDs^32^.

In this literature, a novel g-C_3_N_4_/SnO_2_/WO_3_QDs (denoted as CNSWQ) nanocomposite with full-spectral response was successfully synthesized. Components ratios of the nanocomposite (urea, SnO_2_, and WO_3_QDs solution) were optimized based on response surface methodology (RSM). The photocatalytic degradation of raw landfill leachate by CNSWQ was evaluated under visible-NIR light and sunlight irradiations. Gas chromatography-mass spectrometry (GC–MS) analysis was used to investigate the degradation of various types of organic contaminants and determine intermediate reaction products during the process. Furthermore, the as-prepared photocatalyst demonstrated acceptable stability for the photodegradation of the landfill leachate in multiple cycles. A cost-efficiency analysis was also performed to demonstrate the feasibility of the process.

## Materials and methods

### Materials

Urea and sodium tungstate dehydrate were purchased from Loba, India. Tin dichloride dehydrate (SnCl_2_·2H_2_O) was obtained from Sigma-Aldrich Chemical Co. All other chemicals were bought from Merck Company.

The leachate samples were obtained from the refuse landfill of Kahrizak in Tehran, Iran. Samples were kept in the refrigerator at 4 °C to prevent biological degradation. To remove particles, leachate samples were centrifuged, and then characterized (see Appendix A, Table 1).

### Preparation of SnO_2_ nanoparticles

Synthesis of SnO_2_ photocatalyst was easily done by the vapor hydrolysis method. Initially, 3 g of SnCl_2_·2H_2_O and 20 mL of ethylene glycol were mixed in a glass bottle and stirred for half an hour. The glass was then placed into a Teflon-lined autoclave containing 20 mL of deionized water and placed into an oven at 160 °C for 16 h. After cooling to room temperature, the resulting precipitates were collected and washed several times with ethanol and deionized water and dried at 65 °C overnight.

### Preparation of WO_3_QDs solution

To synthesize WO_3_, firstly, 4.8 g of Na_2_WO_4_·2H_2_O was mixed with 120 mL of distilled water and stirred, followed by adding 12 mL of HCl solution dropwise. Then, after 2 h ultra-sonication and 1 h of stirring, the product was centrifuged and washed with ethanol and water several times and dried at 80 °C overnight. To synthesize WO_3_QDs, 3 g of obtained WO_3_ powder was taken in 450 mL of distilled water and stirred for half an hour. The well-dispersed suspension, then, was sonicated for 20 h. After centrifugation at 10,000 rpm for 10 min, WO_3_ QDs solution with a concentration of 3 g/L was obtained, which is 30 times higher than the value obtained in the similar previous study^33^.

### Preparation of g-C_3_N_4_/SnO_2_/WO_3_QDs nanocomposite

To prepare the g-C_3_N_4_/SnO_2_/WO_3_QDs nanocomposite, 14 g urea was added to a round-bottom flask in an oil container which was placed on a stirrer hot plate with a temperature of 240 °C. After urea was melted, 205 mg synthesized SnO_2_ and 13 mL WO_3_ QDs solution was added to the flask. Then, the mixture was stirred for 3 h and moved to a crucible with a cover. The crucible was put in a muffle furnace with a temperature of 550 °C for 2 h. Finally, the obtained g-C_3_N_4_/SnO_2_/WO_3_QDs was grounded into powder after cooled down to room temperature.

### Characterization

The structure of CN, SnO_2_, g-C_3_N_4_/SnO_2_ (CNS), and CNSWQ was specified by X-ray diffraction (XRD) measurement with a PW1730 XRD instrument (The Netherlands). Scanning Electron Microscope (SEM) and Energy Dispersive X-ray (EDX) were applied to the image and analyze the morphology and microstructure of as-prepared samples and to determine the crystallite size and the mixing ratio of particles by an acceleration voltage of 30 kV and SAMX detector (Tescan, Mira3, Czech Republic). The transmission electron microscopy (TEM) analysis was done to analyze the nanoparticles with high magnification on a CM120 (The Netherlands) transmission electron microscope operating. Photoluminescence (PL) spectra were determined by a TEC 2048 Avaspec (The Netherlands) photoluminescence spectrophotometer at room temperature with an excitation wavelength of 325 nm. The UV–Vis diffuse reflectance spectra (UV–Vis DRS) were measured by a Scinco S4100 (South Korea) spectrophotometer. The energy gap (Eg) of CN, CNS, and CNSWQ is determined using the following equation^34^.

1 $$ {\text{E}}_{{\text{g}}} \left( {{\text{eV}}} \right) = {124}0/\lambda $$

where E_g_ is the energy gap (eV), and λ is the wavelength of absorption edge (nm). Fourier transform infrared (FTIR) spectroscopy was carried out with an FTIR spectrometer (America Perkin Elmer, Spectrum 65) using the standard ASTM E168. Brunauer, Emmett and Teller (BET) method surface area instrument (Belsorp mini Japan) was utilized to investigate the surface area of the samples.

### Evaluation of photocatalytic performance

**Figure 1:**
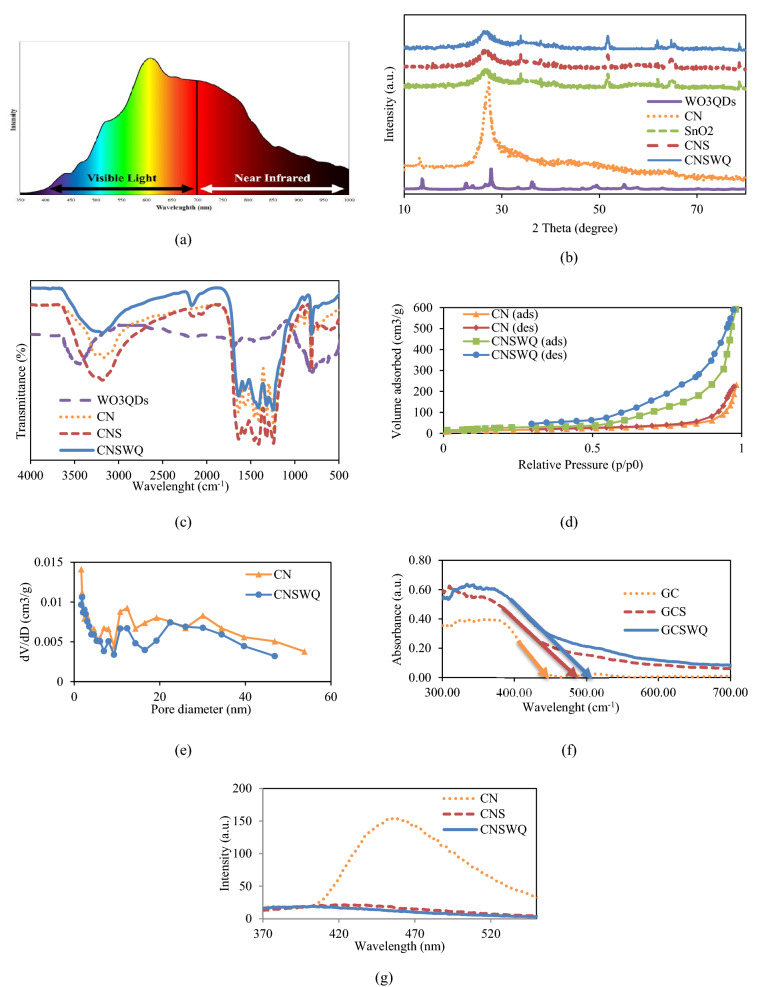
(**a**) Emission spectra of the lamp, (**b**) XRD, (**c**) FTIR spectra, (**d**) N_2_ adsorption–desorption curves, (**e**) BET surface area, (**f**), UV–Vis spectra, and (**g**) PL spectra of the photocatalysts.

The photodegradation of landfill leachate was carried out under a 150 W Osram HLX 64633 halogen lamp with a wide wavelength, as shown in Fig. 1a. Typically, 0.25 g of CNSWQ photocatalyst was dispersed in 100 mL of landfill leachate and stirred for 240 min. In order to measure the degradation of the leachate, samples were centrifuged to separate the catalysts and analyzed by the DR-3900 Hach spectrophotometer. Similar processes without irradiation and photocatalyst were performed for 1 h to measure the impact of each parameter individually.

### Use RSM to determine the best mixing ratios and interactions of nanocomposite components

RSM was used to evaluate and optimize the individual and interaction effects of three key synthesis parameters of the g-C_3_N_4_/SnO_2_/WO_3_QDs photocatalyst: urea, SnO_2_ nanoparticles, and WO_3_ solution. These parameters were considered independent variables, and the chemical oxygen demand (COD) removal of the leachate (percentage) after 1 h of irradiation was regarded as the response (dependent variable). To determine the impacts of the three independent variables on the response, experimental levels of independent variables were determined (see Appendix A, Table 2), and a central composite design (CCD) was employed using 20 sets of experiments.

### Feasibility and financial calculation

The feasibility of the photodegradation process is related to the cost of treatment per unit COD of the wastewater. The operating expenditures (OPEX) of photocatalytic purification include the cost of energy consumption (CEC) and the cost of chemicals and synthesizing (CCS). In this study, the CCS was not formulized because of the presence of a wide variety of photocatalysts and synthesizing methods that vary with the geographic locations, equipment, and materials and is unique in each case. The OPEX could be determined from Eqs. (2) to (4) as follows^35^:

2 $$ {\text{OPEX}}\left( {{\text{USD}}/{\text{g}}_{{{\text{cod}}}} } \right) = {\text{CCS}} + {\text{CEC}} $$

3 $$ {\text{SEC}}\left( {{\text{kW}}\;{\text{h}}/{\text{g}}_{{{\text{cod}}}} } \right) = \frac{{{\text{P }} \times {\text{T}}}}{{{\text{V }} \times { }\left( {C_{0} - C_{f} } \right)}} $$

4 $$ {\text{CEC}}\left( {{\text{USD}}/{\text{g}}_{{{\text{cod}}}} } \right) = {\text{SEC}} \times 0.{14}\left( {{\text{USD}}/{\text{kW}}\;{\text{h}}} \right) $$

where SEC: specific energy consumption, P: the electric power of the photochemical system (kW), T: the reaction time (h), V: the volume of the solution in the reactor (L), C_o_: the initial concentration of each organic compound (g/L), C_f_: the final concentration of each organic compound (g/L).

## Results and discussion

### Structural analysis of the synthesized photocatalysts

Figure 1b represents the XRD patterns for the WO3 QDs, CN, SnO_2_, CNS, and CNSWQ. The XRD pattern of the WO_3_ QDs corresponds with the pure WO_3_ hexagonal structures (JCPDS card No. 85-2460) and demonstrates the sample crystalline structure. The diffraction angles of 13.82, 22.8, 24.31, 27.88, 36.31, 49.5, 55.04, are ascribed to (100), (002), (110), (200), (202), (302), and (302) planes, respectively^36^. The bare CN catalyst exhibits two well-defined diffraction peaks at 13.04° and 27.40°, corresponding to (100) and (002) diffraction planes, respectively (JCPDS No. 87-1526)^37^. The main diffraction peaks of the as-prepared SnO_2_ are distinguishable at 26.58°, 33.88°, 37.95°, 51.75°, 61.89°, 64.73°, and 78.69°, which could be related to (110), (101), (200), (211), (310), (112) and (321) planes of SnO_2_ (JCPDS No. 25-0922)^38^. In XRD patterns of CNS, no obvious CN peaks could be detected, which might be because of the small amounts of CN layers in the CNS nanocomposite. Furthermore, the close location of the main CN peak at 27.40° and the SnO_2_ peak at 26.58° makes it difficult to distinguish one peak from the other^39^. Also, no considerable peak of WO_3_ crystals could be detected. It might be due to the very tiny particle sizes of WO_3_ QDs^40^. According to the Scherrer equation, D = kλ/(βcosθ), where D is the particle size, k is the so-called shape factor (0.9), λ is the X-ray wavelength (0.15406 nm), β is the full width at half maximum intensity in radians and θ is the Bragg angle. The calculated overall particle size of the photocatalyst was about 13 nm. Moreover, the average particle size of WO_3_ QDs was calculated to be 8.4 nm.

The presence of the expected components of WO_3_ QDs, CN, CNS, and CNSWQ was investigated by FTIR spectra and displayed in Fig. 1c. For WO_3_ QD, peaks at 632, 810, and 923 are ascribed to the stretching vibrations of W–O–W, bending vibrations of W–O–W and O=W, and bending vibrations of W–O–W, respectively. It can be concluded that the WO_3_ nanostructures are well formed. Moreover, the detected absorptions at 3438 may be attributed to the bending and stretching vibration of –OH^41^. An absorption peak at 812 cm^−1^ and a wide absorption band with a range from 1238 to 1651 cm^−1^ are significant in FTIR spectra of CN, CNS, and CNSWQ, ascribing to stretching vibrations of g-C_3_N_4_ heterocyclic moieties and breathing mode of s-triazine units and the peak at 1649 cm^−1^ is related to the C=N stretching vibration modes^24,42^. Furthermore, the known peaks at 737 cm^−1^ and 812 cm^−1^ in CNS can be attributed to the anti-symmetric and symmetric O–Sn–O stretching, demonstrating the excellent combination of g-C_3_N_4_ and SnO_2_^43^. In addition, In the FTIR spectra of CNSWQ, the vibration peak of the s-triazine ring at 812 cm^−1^ was a little shifted, which demonstrated that nitrogen pots may be structurally deformed because of the interplay of g-C_3_N_4_ and WO_3_QDs^44^.

N_2_ adsorption and desorption isotherms were used to investigate the BET surface area, pore size, and pore distribution of the as-prepared photocatalyst. The BET surface area and Barett–Joyner–Halenda (BJH) pore size distribution of CN and CNSWQ are illustrated in Fig. 1d,e. Both samples displayed the type IV isotherms, which demonstrates the mesoporous formation with relative pressure in the range of 0.1–0.98. The specific surface area for bare CN and CNSWQ photocatalysts are 55.258 and 257.54 m^2^/g, respectively, and the average pore size diameter of CN and CNSWQ are 25.939 nm and 21.603 nm. The specific surface area of CNSWQ nanocomposite is larger than CN, which indicates that CNSWQ has more active sites and enhanced absorption and degradation ability compared to bare CN.

The spectral characteristics of the as-prepared CN, CNS, and CNSWQ were investigated using diffuse reflectance spectra. As depicted in Fig. 1f, CN, CNS, and CNSWQ can absorb UV and visible lights because of their narrow band gaps. Furthermore, CNSWQ nanocomposite exhibited a considerable absorption tail in the 700 nm boundary, indicating a great absorption capacity in the Vis–NIR light region. It could be attributed to the metal-like LSPR induced with the collective oscillation of free electrons on the surface of WO_3_ because of the plentiful oxygen vacancies^44^. The absorption edge of CN, CNS, and CNSWQ lie at 445, 484, and 502 nm, respectively. The band gap of CN was estimated to be 2.78 eV, while the E_g_ of CNS was calculated to be 2.56 eV. This might be because of the synergistic effects between CN and SnO_2_ that can cause the promotion of photogenerated charge carriers separation and visible light absorption^24^. The bandgap energy of CNSWQ was also calculated to be 2.47 eV. This enhancement of UV–Vis-NIR light absorption might be due to improved separation of the photoinduced electrons and the LSPR effect of the WO_3_QDs^44^.

PL spectra analysis of CN, CNS, and CNSWQ that mainly correlated with the recombination rate of photogenerated electrons and holes is depicted in Fig. 1g. It is clear that the CN sample showed a broad emission band in the 400–550 nm region and has a strong emission band centered at about 460 nm because of the recombination of the photoinduced electron–hole pairs. Compared with bare CN, the band intensity of photoluminance spectra for CNS decreased notably, demonstrating that combination with SnO_2_ could impede the fluorescence of CN. Moreover, the PL emission intensity of CNSWQ was reduced in comparison with CNS, indicating that after the assembly of WO_3_QDs on the CN nanosheet surface, the recombination of the photoinduced charge was considerably inhibited.

**Figure 2:**
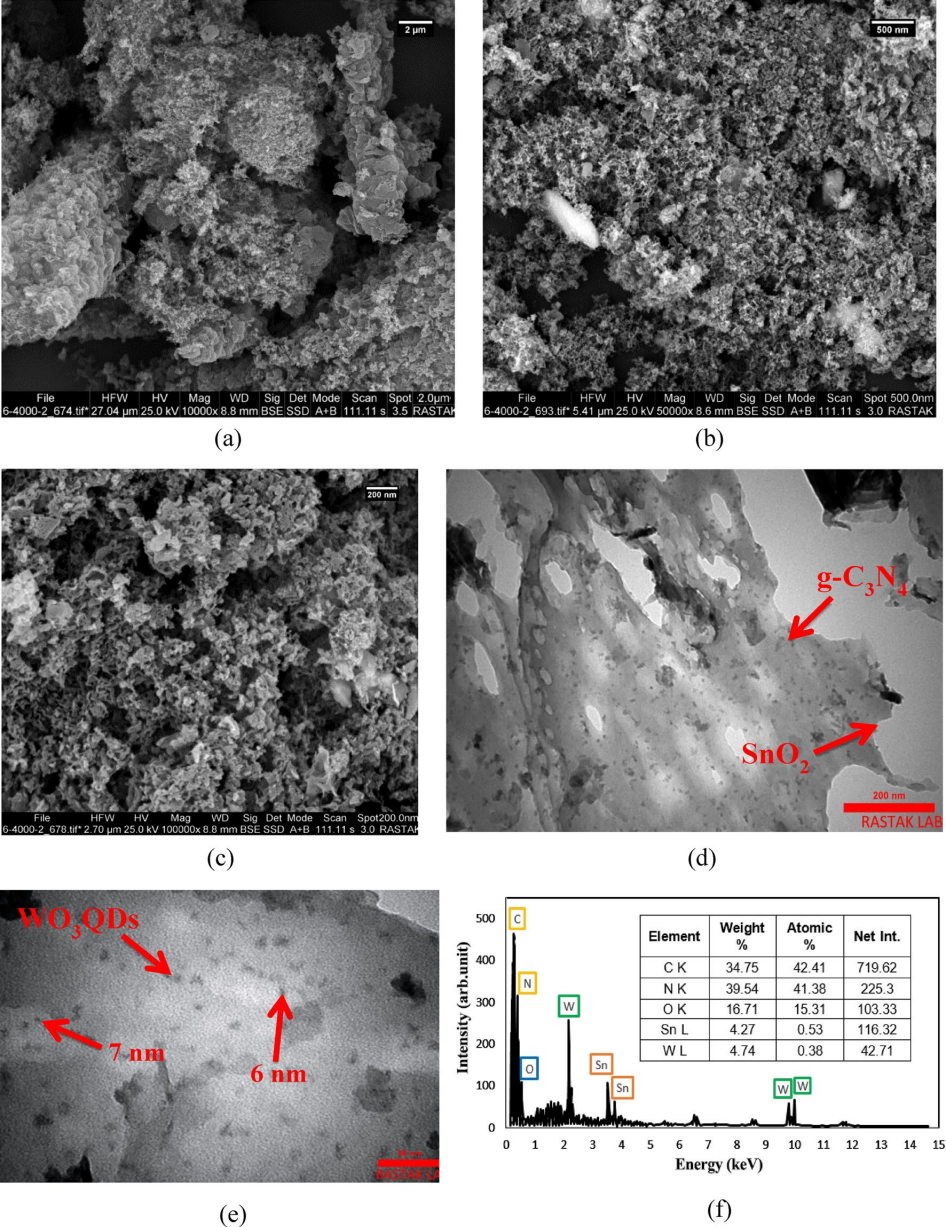
SEM images of (**a**) CN, (**b**) CNS, (**c**) CNSWQ, and (**d**,**e**) TEM images of CNSWQ, and (**f**) EDX analysis of CNSWQ.

SEM observations of the photocatalysts are represented in Fig. 2a–c. In Fig. 2a, it is clear that synthesized CN has a lamellar structure with a rough and porous surface. It is because of thermal exfoliation, which is due to internal stresses between the layers by applying temperature. Figure 2b,c represents the SEM images of CNS and CNSWQ nanocomposites, respectively. As can be seen, SnO_2_ nanoparticles are evenly scattered on g-C_3_N_4_ layers in all structures. WO_3_QDs cannot be seen in SEM images because of the very small size of the particles. Also, after SnO_2_ and WO_3_QDs were loaded on g-C_3_N_4_ sheets, no significant change in the rough and porous morphology of g-C_3_N_4_ layers was observed. It is noted that the rougher and irregular surface of nanocomposite can enhance pollutant molecules’ adsorption and photodegradation of contaminants^45^.

Figure 2f shows the EDX analysis of CNSWQ photocatalyst, confirming the existence of expected elements. Strong peaks of C, N, Sn, O, and W can be clearly seen in the spectrum, confirming the successful synthesis of the nanocomposite. It also can be noted that there are no unknown peaks which that demonstrate the high purity of the nanoparticles.

To get an in-detailed analysis of nanocomposite structure for better insight, TEM analysis is essential. Figure 2d,e displays TEM images of CNSWQ. The CN nanosheets can clearly be observed, and it is apparent that SnO_2_ nanoparticles are well anchored on the graphitic carbon nitride nanolayers. Furthermore, the TEM images of the nanocomposite indicate that WO_3_QDs are uniformly distributed on the surface of CN, and the size of the quantum dots was found to be less than 10 nm.

### Application of DOE in optimization of the photocatalytic performance

Design Expert^®^ software (Ver. 12) was used to design the best mixing ratio of nanocomposite components to achieve the best removal rate of the leachate COD. The amount of urea (g), SnO_2_ (mg), and WO_3_QDs solution (mL) were the three influencing variables that were investigated by the RSM-CCD method. The complete design matrix was obtained, and 20 runs were performed (see Appendix A, Table 3). Also, to avoid lurking phenomenon, experiments were done randomly and in 2 days (blocks).

The experimental results were fitted to a quadratic model. Equation (5) represents the model of COD removal performance (Y) as a function of urea (X_1_), SnO_2_ (X_2_), and WO_3_QDs solution (X_3_) as follows:

5 $$ {\text{Y}} = {1}0.{13} + {1}.{\text{12X}}_{{1}} + 0.{\text{1383X}}_{{2}} - 0.0{\text{592X}}_{{3}} {-}0.{\text{1362X}}_{{1}} {\text{X}}_{{2}} + 0.{\text{4463X}}_{{1}} {\text{X}}_{{3}} + 0.{\text{3438X}}_{{2}} {\text{X}}_{{3}} {-}0.{\text{6692X}}_{{1}}^{{2}} - 0.{\text{9971X}}_{{2}}^{{2}} - 0.{\text{6523X}}_{{3}}^{{2}} $$

To validate the adequacy of the model, the analysis of variance (ANOVA) was used (see Appendix A, Table 4). Based on the high F-value of the model (F_model_ = 59.85) and the low P value of the model (P_model_ < 0.0001), it could be concluded that the mathematical model is significant. No notable difference between pure error and residual could be seen due to the lack of fit. R^2^, R^2^_adjasted_ and R^2^_predicted_ values obtained 0.9836, 0.9671 and 0.8566, respectively. The value of R^2^ is high enough, and the difference between R^2^_adjasted_ and R^2^_predicted_ is lower than 0.2, demonstrating the adequacy of the model^46–48^.

**Figure 3:**
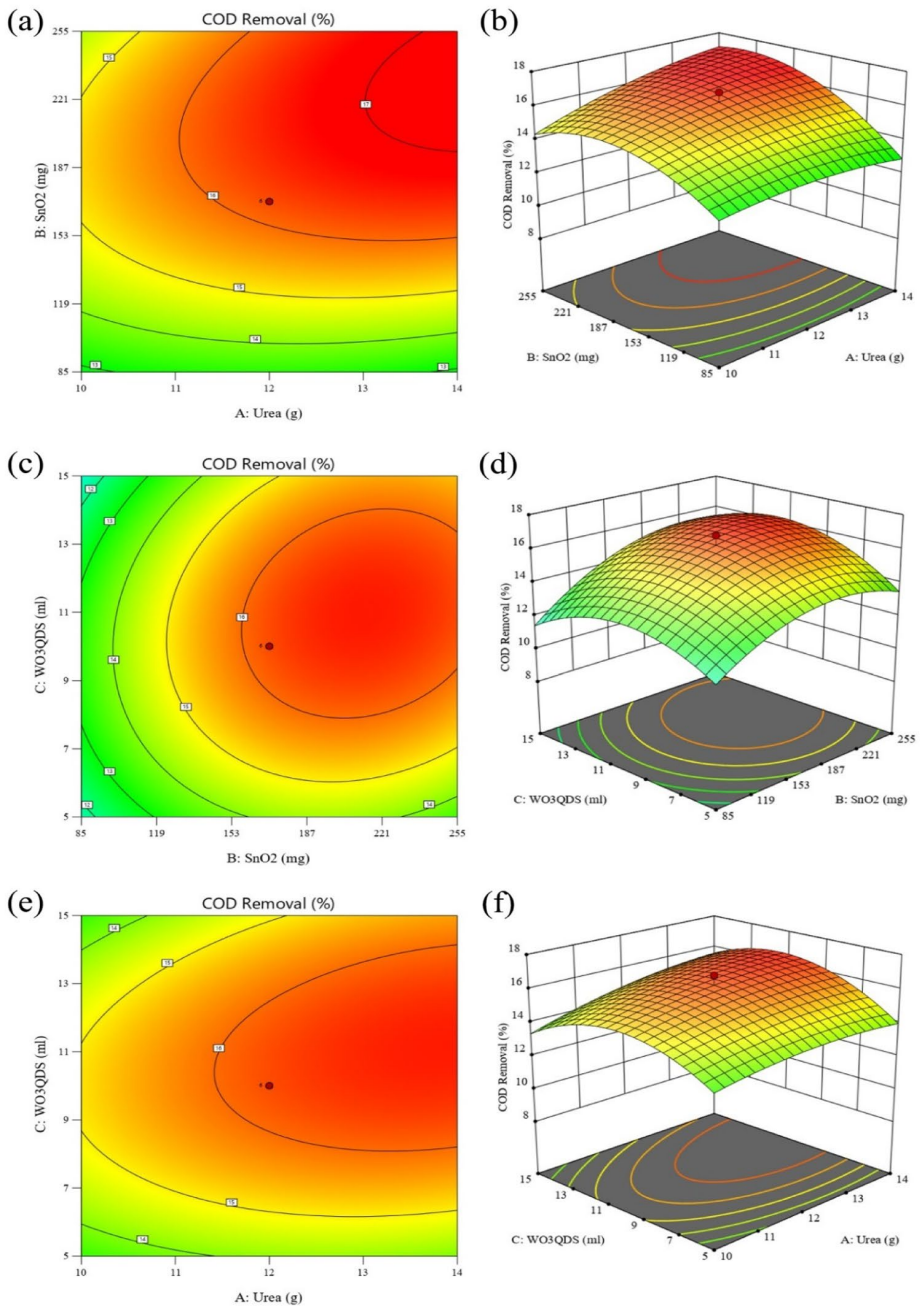
3D response surface graphs and contour plots for leachate photodegradation: (**a**,**b**) SnO_2_ vs. urea, (**c**,**d**) SnO_2_ vs. WO_3_QDs solution, and (**e**,**f**) urea vs. WO_3_QDs solution.

Figure 3 represents the two-dimensional contours and the 3D response surface plots of the effect of interactions between parameters on COD removal of the landfill leachate. As can be seen in Fig. 3a,b, the COD degradation of leachate increased by increasing the ratio of SnO_2_ to urea. The synergistic effect between SnO_2_ and CN can cause a boost in charge separation and charge transfer and decreases charge recombination; as a result improving the photodegradation process. It can be noted that this is entirely consistent with the results obtained from the photoluminescence spectra test. As displayed in Fig. 3c,d, COD removal of the landfill leachate showed a trend of first increasing and then decreasing along with the increment of SnO_2_ nanoparticles amount. The effect of the amount of WO_3_QDs solution on COD removal also displayed the same trend. Moreover, Fig. 3e,f illustrates the interaction impact of urea and WO_3_QDs solution on the degradation of the landfill COD. As it is shown, the maximum photodegradation of the leachate COD occurred at higher amounts of urea, but by increasing the amount of WO_3_QDs solution, the COD removal trend first increased and then decreased. This increment could be due to the enhancement of the charge separation efficiency because of the LSPR effect and the heterojunction formation. Lower or higher amount of WO_3_QDs solution exhibited a lower photodegradation due to non-optimal synergistic impact between the g-C_3_N_4_ and WO_3_QDs (lower amount of WO_3_QDs solution) by covering the active sites of g-C_3_N_4_ by WO_3_QDs (higher amount of WO_3_QDs solution)^44^.

**Figure 4:**
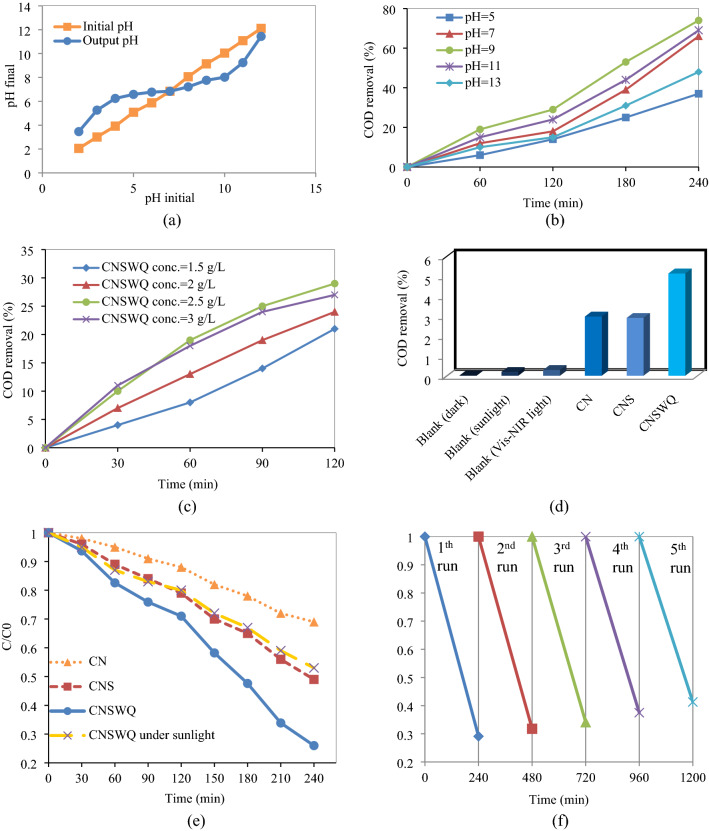
(**a**) pH_PZC_ of CNSWQ and effect of (**b**) pH, (**c**) photocatalyst dosage, (**d**) adsorption (dark), Vis–NIR light (alone), sunlight (alone), (**e**) photocatalysts under Vis–NIR lamp and sunlight irradiation, and (**f**) cycling runs on COD degradation of landfill leachate.

The suitability of the model could be demonstrated by diagnostic plots. The predicted vs. actual plot and the residuals vs. predicted plot are represented in Fig. 1a,b of Appendix A. The association of actual and predicted responses represents a low deviation from the diagonal line. A good agreement between actual data and the ones that were achieved from the models can be seen. The difference between the predicted responses and experimental responses is depicted in Fig. 4b, displaying a random normal distribution of residuals. The randomly distributed residuals without any trends represent acceptable predictions of maximum response along with constant variance and show the adequacy of the quadratic model.

The contribution percentage of each parameter and interactive parameters is illustrated in Fig. 1c,d of Appendix A. As it is shown, the individual effect of SnO_2_ on photodegradation of the COD of the landfill leachate by a contribution percentage of 76% is the highest among the other parameters. Also, urea and WO_3_QDs have individual effects of 18 and 6 percent of the contribution. The effect of interactions between urea and SnO_2_, SnO_2_ and WO_3_QDs, and urea and WO_3_QDs are 50%, 31%, and 19%, respectively. The interactions between urea and SnO_2_ have the highest impact on the photocatalysis process, which has conformity with similar previous studies. Finally, the best amounts of parameters (in range) to achieve the maximum removal rate of the leachate COD with desirability of 1 were obtained as follows: urea  = 13.654 (g), SnO_2_ = 205.435 (mg), and WO_3_QDs solution = 12.811 (mL). The predicted COD removal using these amounts was obtained 17.087 after 1 h, which completely corresponds to the values obtained from the experiments.

### Photocatalytic performance

pH can significantly affect photocatalytic degradation and is considered an effective parameter of the process due to its impact on factors such as oxidation and reduction potential, surface charge, and position of energy bands of nanoparticles^49^. COD removal of the Determining the point of zero charge (pH_PZC_) is essential to anticipate the surface of the nanoparticles during the photodegradation process, and it is an effective factor in the adsorption rate of the nanocomposite. The pH_PZC_ of the CNSWQ is shown in Fig. 4a. The pH_PZC_ is the point where the curve of Output pH vs. Initial pH intersects the line Output pH = Initial pH. The value obtained for the pH_PZC_ of the CNSWQ photocatalyst was 6.84. As the pH value of the leachate solution is more than obtained pHpzc of the nanocomposite, the CNSWQ surface is negatively charged and promotes the removal of cationic pollutants as a result of the enhanced electrostatic force of attraction^50^. Landfill leachate at different pHs using 250 mg of g-C_3_N_4_/SnO_2_/WO_3_QDs nanocomposite in 240 min is shown in Fig. 4b. The maximum COD degradation was obtained at pH 9. As can be seen, pHs less or more than 9 can reduce the COD removal of the leachate. One explanation might be that this pH maximizes the formation of hydroxyl radicals mainly by preventing the recombination of the electron–hole pairs^51^. In addition, as the best pH value is higher than the pH_PZC_, it can be concluded that most of the leachate contaminants are positively charged, as it effectively helps the adsorption process of the nanocomposite^52^. Moreover, as deprotonation increases the electron density on the contaminants, which promotes the electrophilic attack of reactive oxygen species (like hydroxyl radicals), the quinoline ring at pH 9 may be more susceptible for the hydroxyl radicals attack^53^.

As it is shown in Fig. 4c, COD degradation significantly enhanced by increasing the concentration of CNSWQ photocatalyst up to 250 mg in 100 mL of the leachate due to increasement in the generation of hydroxyl radicals. By increasing the amount of nanocomposite to more than 250 mg, no significant enhancement of COD removal was observed, which may be due to the flocculation of nanoparticles, reduction of the active holes available on the catalyst surface, and increase of light diffraction.

As it is shown in Fig. 4d, after 60 min and without any photocatalysts, the COD of the leachate was only reduced by 0.18% and 0.28% under Vis–NIR and sunlight irradiations, respectively. Furthermore, when the CN, CNS, and CNSWQ were added and stirred in the reactor without any light, the COD of the landfill leachate was reduced by 2.96%, 2.89%, and 5.12%, respectively, due to enhanced adsorption of the nanoparticles. Figure 4e illustrates the visible-NIR light assisted landfill leachate COD removal curves of C/C_0_ versus time during the photodegradation process with CN, CNS, and CNSWQ photocatalysts with the best mixing ratios obtained by response surface methodology. The decomposition reactions are assumed to be correlated to hydroxyl radical's reactions which are made by multiplex reactions of photogenerated charge carriers with water, oxygen, and hydroxide ions. The total photodegradation of leachate COD approached 51% at 240 min for CNS nanocomposite under visible-NIR light irradiation, while removal of leachate COD for an equivalent time (240 min) with bare CN was about 31%, which might be a result of the boosted charge separation and the greater surface area^54^. Eventually, CNSWQ displayed the highest photocatalytic performance and reduced about 74% and 47% of landfill leachate COD under visible-NIR light and sunlight irradiations, respectively. As it can be seen, the addition of WO_3_ quantum dots to the nanocomposite caused a significant enhancement in photocatalytic efficiency, which could be ascribed to the LSPR impact and the formation of heterojunction, and the promotion of charge separation performance^44^. Factors such as adsorption and light (alone) can affect the results of photocatalytic reactions, making it necessary to evaluate the impact of each parameter separately. To fulfill this aim, the effects of these parameters on the system were examined independently. A comparison of landfill leachate photodegradation efficiency between current work and previous studies (Table 1) demonstrates the high performance of optimized g-C_3_N_4_/SnO_2_/WO_3_QDs in the treatment of highly contaminated raw leachate.

**Table 1:** Comparison of landfill leachate photodegradation, CEC, and other properties of current work and other studies.

Leachate COD (mg/L)	Raw leachate	Photocatalyst	Optimized amounts	Energy source (W)	Retention time (h)	Reactor volume (L)	Photocatalyst mass (g)	COD removal (%)	CEC (USD/g_COD_)	References
550	No	W–C-TiO_2_	No	40 (LED light)	40	1.925 (1.5 L of leachate)	0.6354	84	0.27	^62^
1000	No	AC/TiO_2_	No	32 (UVA)	1	0.2	3	48.75	0.05	^63^
800	No	Commercial ZnO	No	32 (UVC)	4	1	2.4	61	0.04	^56^
1135	Yes	Commercial ZnO	No	Sunlight	5	0.5	1.2	50	–	^64^
600	No	W-TiO_2_	Yes	72 (fluorescent)	34	0.25 (0.15 L of leachate)	0.3	46	8.28	^65^
4575	Yes	CNSWQ	Yes	150 (Vis–NIR)	4	0.1	0.25	74	0.25	Current study

One key parameter for the feasibility aspect is the lifetime of the photocatalyst. Therefore, it is essential to evaluate the photocatalytic stability and recyclability of the nanocomposite during several cycles of photodegradation. To do so, after each cyclic run, the sample was centrifuged, and the photocatalyst was washed several times. Then it dried out to become ready for the next run. As shown in Fig. 4f, the stability and recyclability of CNSWQ were assessed by conducting five cycles of experiments for the removal of leachate COD. As can be seen, a slight decrease in the photodegradation of leachate occurred, which may be because of some losses of the photocatalyst during recovery, and no significant loss of degradation performance could be seen. It demonstrates the acceptable stability and recyclability of CNSWQ for degradation of pollutants after many cycles during the wastewater treatment, which is an essential factor in terms of the feasibility of the process.

### Degradation of contaminants during the process

**Figure 5:**
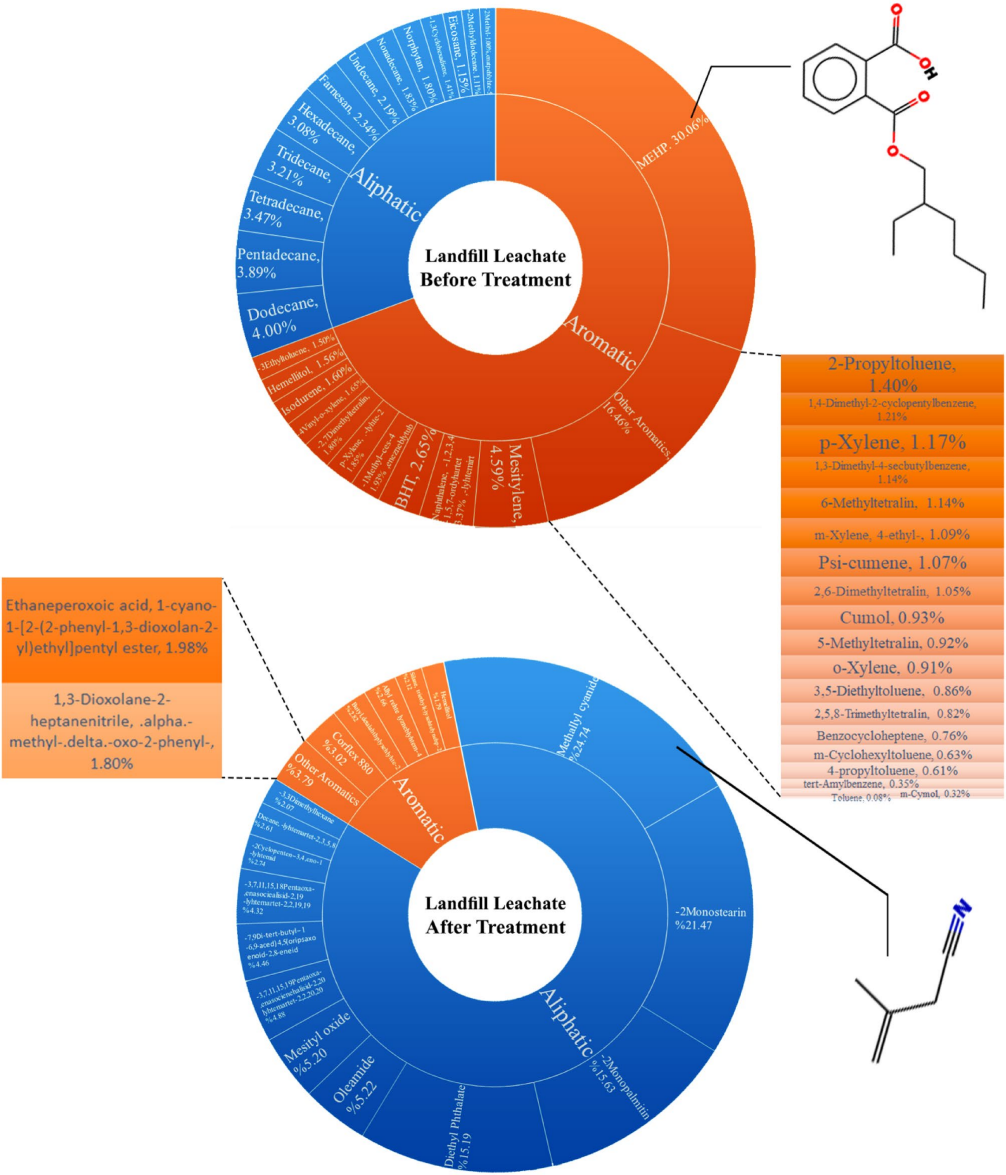
Organic contaminants of the landfill leachate before and after the treatment.

Landfill leachate is one of the major highly toxic pollutants, which often contains a mixture of organic or inorganic contaminants and many refractory compounds. Hydroxyl radicals convert heavy and cyclic molecules of the solution into simpler and smaller molecules that can increase biodegradability through a photocatalytic process^55^. To specify intermediate reaction products, GC–MS analysis was used. Figure 2 of Appendix A shows the GC–MS spectra of the raw leachate and the leachate before and after the photodegradation process. Also, the key aromatic and aliphatic compounds recognized in this analysis using the NIST database are depicted in Fig. 5. The GC–MS spectrum of the leachate after the photodegradation shows peaks at considerably longer retention times than the GC–MS spectrum of the raw landfill leachate, demonstrating a significant reduction of heavier compounds after photodegradation with g-C_3_N_4_/SnO_2_/WO_3_QDs nanocomposite (see Appendix A, Fig. 2)^56^. Figure 5 also demonstrates a remarkable decrease in the number of the leachate pollutants, especially the toxic aromatic compounds, after the treatment process. As it can be seen, MEHP, as a highly toxic material, constitutes a great proportion of the raw leachate contaminants. MEHP is the intermediate biodegradation product of DEHP. DEHP causes many diseases, including cancer, reproductive, developmental, nerve, and immune toxicities, and endocrine disruption effects in rodents^57^. It is shown that PVC plastics contribute to additional MEHP in the landfill leachate^58^. Therefore, considering that the photocatalyst has remarkably degraded MEHP, it can be concluded that CNSWQ can be effectively used in the treatment of wastewaters that contain high amounts of PVC products. On the other hand, Methallyl cyanide, Monostrearin-2, Monopalmitin-2, and Diethyl Phthalate constitute a major part of the leachate contaminants after the photodegradation process. It can be concluded that after the treatment process, volatile compounds of the leachate not only became simpler in structure, but became less toxic. Methallyl cyanide, the compound with the highest proportion in the treated leachate contaminants, is present in flavor rapeseed oil (FRO) which is widely used in multiple cuisines as a key ingredient^59^. Monostrearin-2 and Monopalmitin-2 have simple structures with no aromatic rings. Moreover, Diethyl Phthalate, which is widely used in the industry, has low toxicity to humans and animals^60^.

### Financial analysis

The feasibility of the photocatalytic treatment could be investigated by evaluating the cost of the photodegradation process, so a preliminary cost estimation was performed in this study. At the optimum condition, when the pH is 9, the mass of the photocatalyst is 2.5 g/L, the volume of the reactor is 0.1 L, the retention time is 4 h, and the energy consumed by the 150 W lamp is considered the electric power of the photochemical system, the CEC of the process is 0.25 USD/g_COD_ which is cheaper than the other similar studies on the treatment of leachate under visible light (Table 1). This is despite the fact that g-C_3_N_4_/SnO_2_/WO_3_QDs photocatalyst is also highly active under sunlight, and the cost of energy consumption of the lamp could be avoided, and consequently, the CEC can completely be eliminated. However, due to limitations, some aspects of the possible practical application, such as synthesis, materials, equipment, construction, operation, start-up, and some other direct or indirect costs, were not taken into account in the present work^61^. Although, it is notable that the amount of photocatalyst mass is acceptable compared to other similar studies, which could positively affect the CCS and reduce the cost of chemicals and the synthesis process. Moreover, as stated before, using the presented synthesis method, a WO_3_ QDs solution with 30 times higher concentration (compared to previous studies) can be obtained which leads to a notable decrease in the materials and synthesis costs. Furthermore, urea, which based on the results of RSM and CCD is the main component of the nanocomposite in terms of usage amount, is a low-cost material. However, according to the results of the recyclability tests, the photocatalyst is reusable; thus, if it is properly recovered or immobilized in the system on a practical scale, only the expense of initial preparation or some periodic replacements is left. Therefore, it can be deduced that the photodegradation of landfill leachate using CNSWQ nanocomposite can be considered a cost-effective photocatalytic process.

## Conclusions

In this research, a novel and highly efficient g-C_3_N_4_/SnO_2_/WO_3_ QDs photocatalyst was successfully synthesized, and its properties were studied. The structure of the nanocomposite was identified by XRD and FTIR, and the morphology of g-C_3_N_4_/SnO_2_/WO_3_ QDs photocatalyst was studied using SEM and TEM analyses. Also, based on the results of DRS and PL analyses, the improved photocatalytic activity could be attributed to the strong light adsorption capacity and enhanced separation of electron–hole pairs. The optimized mixing ratios of the nanocomposite components, which were obtained from response surface methodology, resulted in maximum degradation efficiency of 74% and 47% in 4 h towards raw landfill leachate under visible-NIR light and sunlight irradiations, respectively in a pH of 9 and the photocatalyst concentration of 2.5 g/L. Heavier and aromatic compounds of the raw leachate were also significantly reduced after the photocatalysis process, based on the results of the GC–MS spectrum analysis. Furthermore, the photocatalyst showed very good stability and reusability after five multiple cycles of photodegradation. Financial analysis and photocatalytic activity of this nanocomposite under sunlight demonstrated the cost-effectiveness of the process. Therefore, it can be concluded that the g-C_3_N_4_/SnO_2_/WO_3_ QDs photocatalyst could be regarded as a promising photocatalyst, and the development of this nanocomposite could provide a new insight into functional and feasible photodegradation of real wastewaters and pollutants. This research, however, is subject to several limitations. The first is the financial analysis, in which some aspects of the possible practical applications are neglected. The second limitation concerns the characterization analyses. Some techniques which could add more data about the photocatalysts, like selected area electron diffraction (SAED), could not be conducted in this research. The immobilization of the photocatalyst onto various proper beds is recommended for future studies, as it can prevent the separation problems and enhance the photodegradation process, especially in terms of practical applications.

## Supplementary Information

Supplementary Information.

## Data Availability

The datasets used and/or analysed during the current study available from the corresponding author on reasonable request.
