# Supplementary material for: Highly efficient photodegradation of raw landfill leachate using cost-effective and optimized g-C3N4/SnO2/WO3 quantum dots under Vis–NIR light
Source: Sci Rep. 2022 Nov 14;12:19457. doi: 10.1038/s41598-022-24143-3 (PMC9663544; doi:10.1038/s41598-022-24143-3)
Supplement: Supplementary file 1 — Supplementary Information. [file 41598_2022_24143_MOESM1_ESM.docx]

| 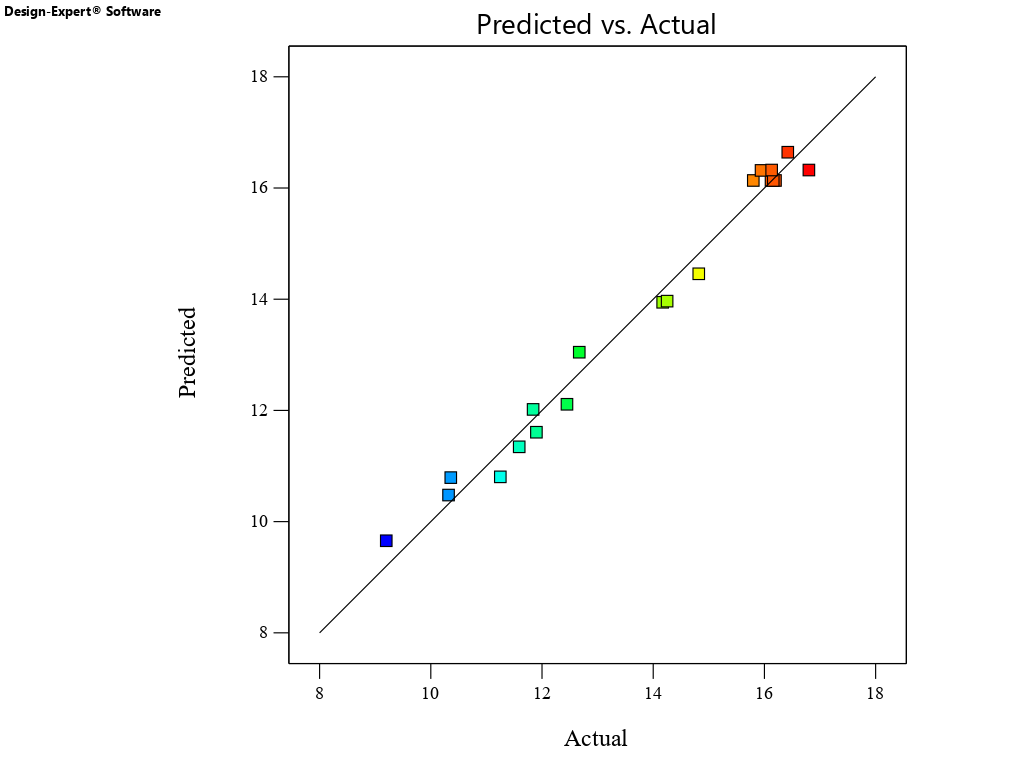 | 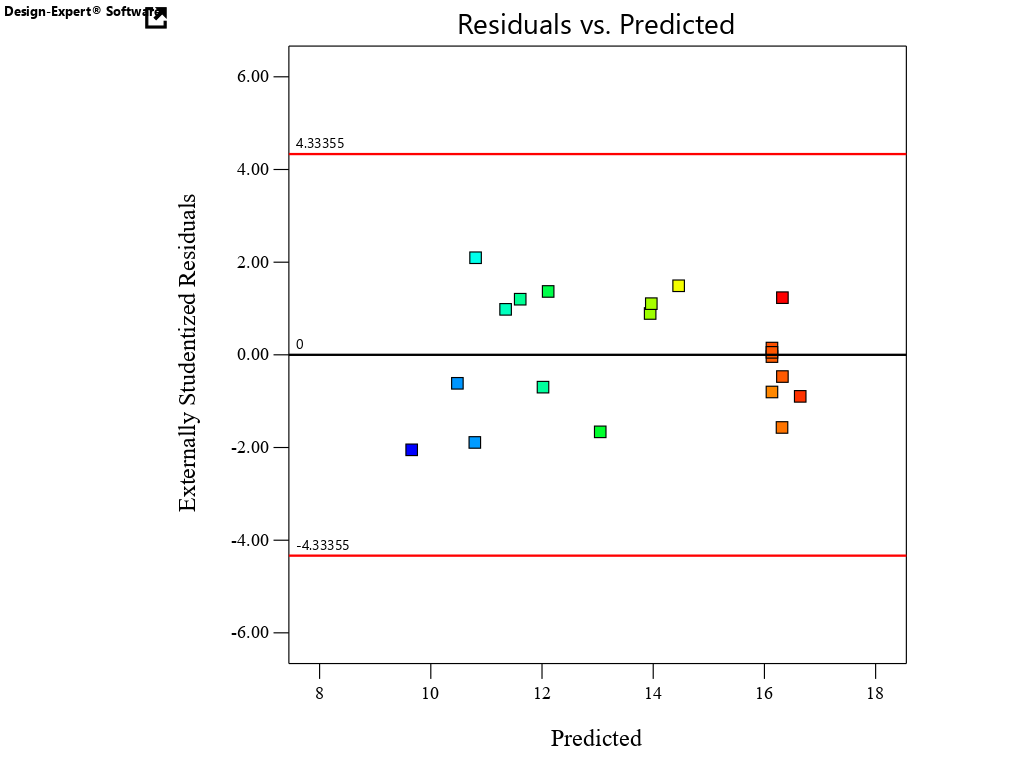 |
| --- | --- |
| (a) | (b) |
|  |  |
| (c) | (d) |

**Fig. 1.** Plots of (a) actual vs. predicted and (b) residuals vs. predicted response values and contribution percentage of (c) individual parameters and (d) interactive parameters

| (a)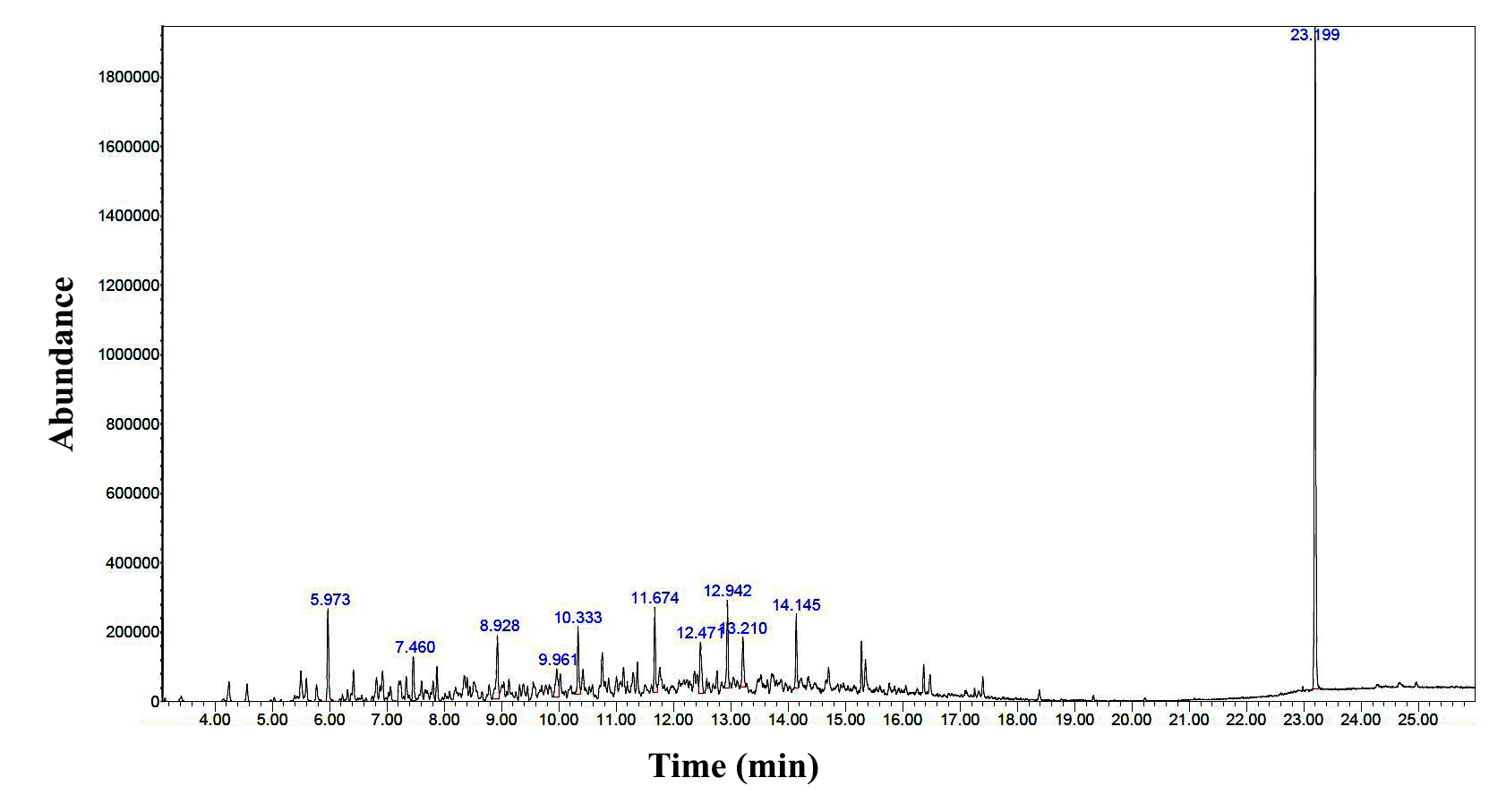 |
| --- |
|  |
| (b)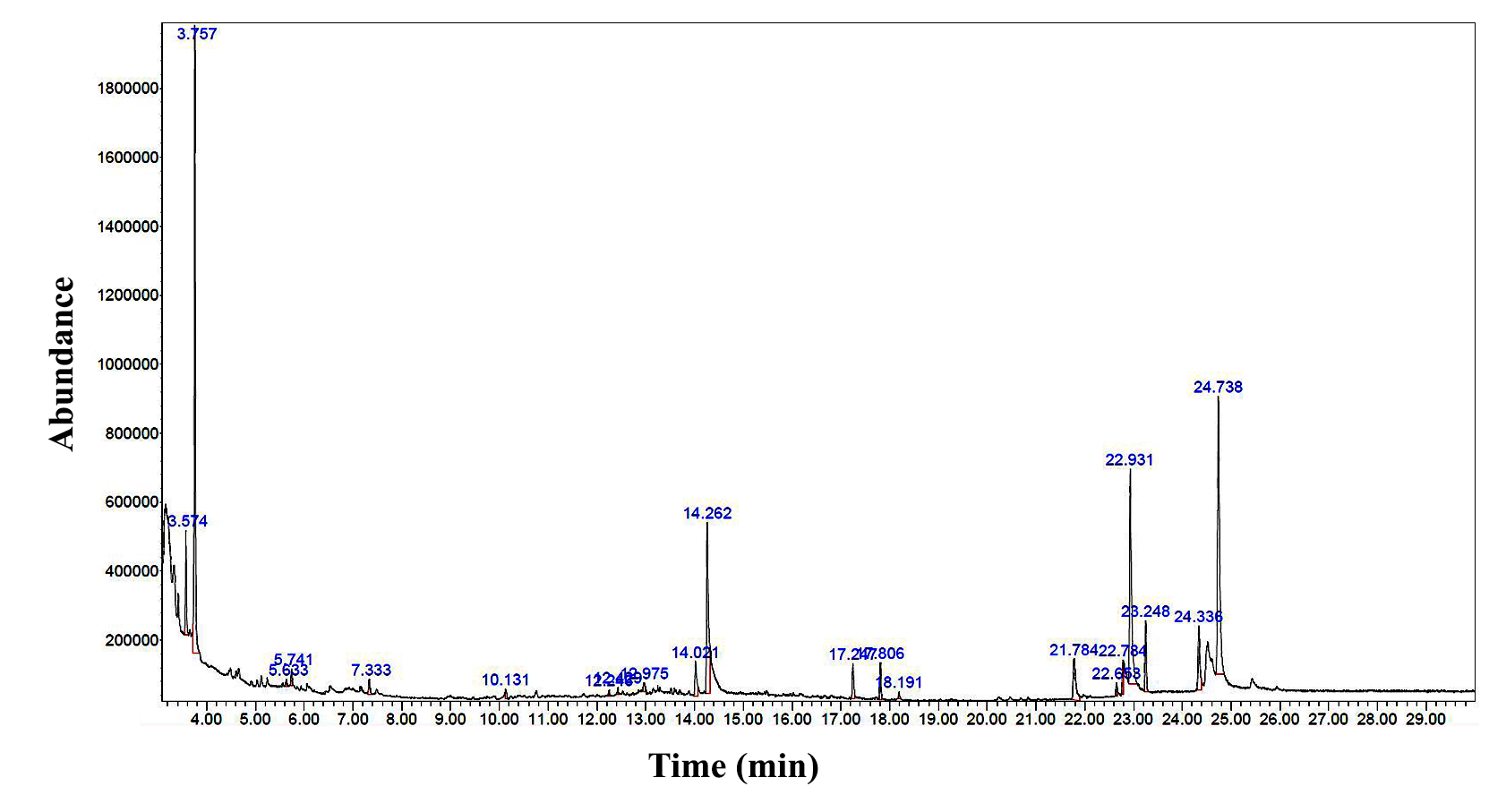 |
|  |

**Fig. 2.** GC-Mass spectrum of leachate (a) before photodegradation process and (b) after photodegradation process under Vis-NIR light irradiation

**Table 1.** Characterizations of the landfill leachate

| Standard Method Code | Value | Unite | Parameter |
| --- | --- | --- | --- |
| - | Dark brown | - | Color |
| - | Liquid | - | Phase |
| 2520C | 0.98 | g/cm^3^ | Density |
| pH meter | 8 | - | pH |
| 5220B  (open reflux method) | 4575 | mg/L | COD |
| 5210B | 835 | mg/L | BOD_5_ |
| - | 0.18 | - | BOD_5_/COD |
| 2540D | 2660 | mg/L | TSS |
| 2540C | 25360 | mg/L | TDS |
| 2540E | 2240 | mg/L | VSS |
| 2520B | 41400 | μS/cm | EC |

**Table 2.** Range and levels of independent variables

| Variable | Symbols | Range and Levels | | | | |
| --- | --- | --- | --- | --- | --- | --- |
|  |  | -1.68 | -1 | 0 | +1 | +1.68 |
| Urea (g) | $X_{1}$ | 8.63 | 10 | 12 | 14 | 15.36 |
| SnO_2_ (mg) | $X_{2}$ | 27.05 | 85 | 170 | 255 | 312.95 |
| WO_3_ QDs (ml) | $X_{3}$ | 1.6 | 5 | 10 | 15 | 18.4 |

**Table 3.** CCD design matrix and results for photodegradation of landfill leachate

| Std | Block | Run | Factor 1 A: Urea  (g) | Factor 2  B: SnO_2_  (mg) | Factor 3  C: WO_3_QDs  (ml) | Response:  COD Removal  (%) |
| --- | --- | --- | --- | --- | --- | --- |
| 11 | Day 1 | 1 | 12 | 170 | 10 | 16.12 |
| 5 | Day 1 | 2 | 10 | 85 | 15 | 10.32 |
| 9 | Day 1 | 3 | 12 | 170 | 10 | 16.2 |
| 2 | Day 1 | 4 | 14 | 85 | 5 | 11.25 |
| 3 | Day 1 | 5 | 10 | 255 | 5 | 11.84 |
| 6 | Day 1 | 6 | 14 | 85 | 15 | 11.59 |
| 8 | Day 1 | 7 | 14 | 255 | 15 | 16.42 |
| 12 | Day 1 | 8 | 12 | 170 | 10 | 16.16 |
| 4 | Day 1 | 9 | 14 | 255 | 5 | 14.17 |
| 10 | Day 1 | 10 | 12 | 170 | 10 | 15.8 |
| 1 | Day 1 | 11 | 10 | 85 | 5 | 11.9 |
| 7 | Day 1 | 12 | 10 | 255 | 15 | 12.67 |
| 16 | Day 2 | 13 | 12 | 313 | 10 | 14.82 |
| 13 | Day 2 | 14 | 8.6 | 170 | 10 | 14.25 |
| 17 | Day 2 | 15 | 12 | 170 | 1.6 | 10.36 |
| 14 | Day 2 | 16 | 15.4 | 170 | 10 | 15.94 |
| 18 | Day 2 | 17 | 12 | 170 | 18.4 | 12.45 |
| 19 | Day 2 | 18 | 12 | 170 | 10 | 16.13 |
| 20 | Day 2 | 19 | 12 | 170 | 10 | 16.8 |
| 15 | Day 2 | 20 | 12 | 27 | 10 | 9.2 |

**Table 4**. ANOVA results for response surface quadratic model of photodegradation of leachate

| Variable | Regression Coefficient | Mean Squares | F-value | P-value | Percentage of Contribution |
| --- | --- | --- | --- | --- | --- |
| Model | - | 12.55 | 59.85 | < 0.0001 | - |
| X_1_ | +0.6987 | 6.67 | 31.79 | 0.0003 | 5.46 |
| X_2_ | +1.43 | 27.82 | 132.63 | < 0.0001 | 22.79 |
| X_3_ | +0.3921 | 2.1 | 10.01 | 0.0115 | 1.72 |
| X_1_ X_2_ | +0.6825 | 3.73 | 17.77 | 0.0023 | 3.06 |
| X_1_ X_3_ | +0.4175 | 1.39 | 6.65 | 0.0298 | 1.14 |
| X_2_ X_3_ | +0.5400 | 2.33 | 11.12 | 0.0087 | 1.91 |
| X_1_^2^ | -0.4181 | 2.52 | 12.00 | 0.0071 | 2.06 |
| X_2_^2^ | -1.51 | 32.78 | 156.27 | < 0.0001 | 26.85 |
| X_3_^2^ | -1.72 | 42.73 | 203.73 | < 0.0001 | 35.00 |
